# Valve-sparing aortic root replacement and aortic valve repair for a 2-year-old girl with Loeys–Dietz syndrome

**DOI:** 10.1093/icvts/ivab367

**Published:** 2021-12-29

**Authors:** Shuichi Shiraishi, Yutaka Okita, Maya Watanabe, Masanori Tsuchida

**Affiliations:** 1 Division of Thoracic and Cardiovascular Surgery, Niigata University Graduate School of Medical and Dental Sciences, Niigata, Japan; 2 Department of Cardiovascular Surgery, Takatsuki General Hospital, Takatsuki, Japan

**Keywords:** Loeys–Dietz syndrome, Valve-sparing aortic root replacement, Aortic valve repair

## Abstract

We report the case of a 2-year-old girl with Loeys–Dietz syndrome complicated by aortic root dilatation and aortic regurgitation. We performed valve-sparing aortic root replacement with reimplantation technique and aortic valve repair using central plication and free-margin reinforcement simultaneously. The postoperative course was uneventful and the latest echocardiography, 5 years after procedure, revealed trivial aortic insufficiency.

## INTRODUCTION

Loeys–Dietz syndrome (LDS) is a rare connective tissue disorder characterized by arterial tortuosity and aneurysm, hypertelorism and bifid uvula or cleft palate. Several reports of valve-sparing aortic root replacement (VSAR) for small children with LDS have been published [1, 2], but description of concomitant aortic valve repair is rare. We describe herein successful VSAR and aortic valve repair for a 2-year-old girl with LDS.

## CASE REPORT

**Figure 1:**
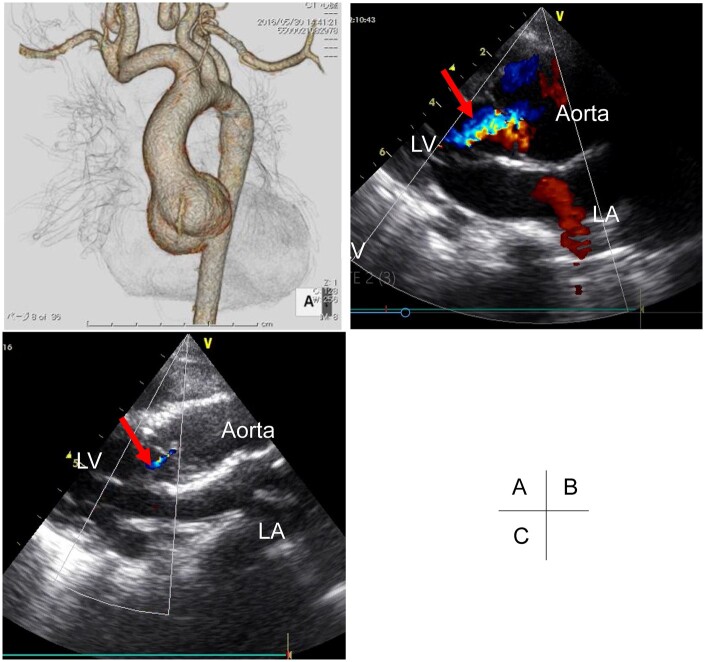
(**A**) Preoperative computedtomography shows dilated sinus of Valsalva. (**B**) Preoperative echocardiography shows moderate aortic regurgitation (red arrow) and right coronary cusp prolapse. (**C**) Postoperative echocardiography shows trivial aortic regurgitation (red arrow). LA: left atrium; LV: left ventricle.

A 1-year-old girl with LDS was refereed to our institution. She had previously undergone surgical closure of an arterial duct at 4 months of age. The ascending aorta and sinus of Valsalva were dilated, with diameters of 18.6 mm (*Z*-score 8.8) and 31.5 mm, respectively. The basal ring was 19 mm (Fig. 1A). On echocardiography, prolapse of the right coronary cusp and eccentric moderate aortic regurgitation (AR) were confirmed (Fig. 1B).

**Figure 2:**
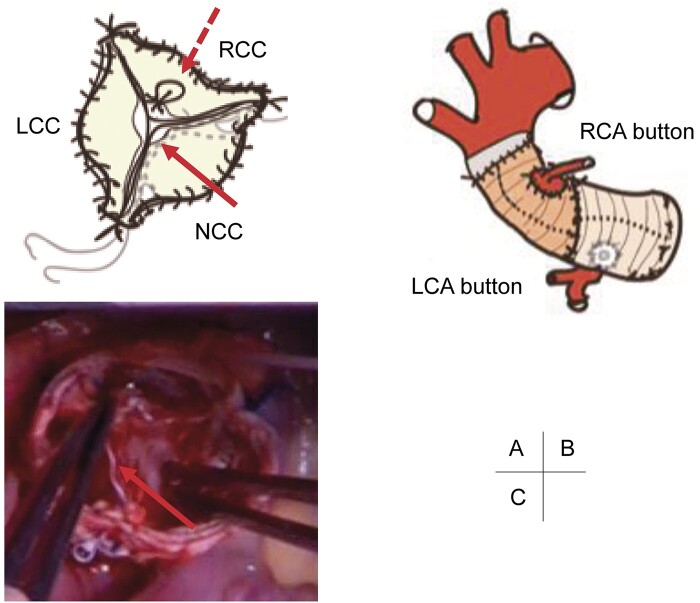
(**A**) Aortic valve repair: central plication of right coronary cusp (dotted arrow); reinforcement of non-coronary cusp (solid arrow). (**B**) Surgical schema. (**C**) reinforcement of NCC (red arrow). LCA: left coronary artery; LCC: left coronary cusp; NCC: non-coronary cusp; RCA: right coronary artery; RCC: right coronary cusp.

We performed VSAR and aortic valve repair when the patient was 2-year-old. Cardiopulmonary bypass was established and the ascending aorta was transected. The aortic valve was balanced tricuspid, and some fenestrations were evident at the non-coronary cusp (NCC). Geometric heights of the right, left and NCCs were 16, 13 and 13 mm, respectively. The aortic root was replaced with reimplantation technique using a 22-mm Gelweave^®^ (Vascutek Terumo Inc., Scotland, UK) straight graft (David-I), and the distal end of the graft was plicated for the purpose of sinotubular junction plication. The graft size of 22-mm was determined using the David’s formula [3]: [diameter = (geometric heights × 2 × 2/3) + (2 × aortic wall thickness) = 20–22 mm], Brussel height of 24 mm, and basal ring of 19 mm. To adjust the height of right coronary cusp, the nodule of Arantius was plicated. The free margin of the NCC was reinforced using CV-7 Goretex^®^ (W. L. Gore & Associates, Inc., Flagstaff, AZ, USA) double-layered mattress running sutures without patch material (Fig. 2). The ascending aorta was replaced with a 16-mm Gelweave^®^ graft. Postoperative echocardiography showed no significant AR. Five years has passed since the operation without any cardiovascular events, and the latest echocardiography revealed trivial AR (Fig. 1C).

### Comment

LDS is an autosomal dominant inherited syndrome caused by heterozygous mutations in the receptors for TGF-b, and aortic dissection and aneurysm rupture occur earlier in LDS patients than in Marfan patients [2]. Fraser *et al.* [4] reported the following indications for VSAR in LDS children: maximal diameter of 3.5–4.0 cm, *Z*-score >3, severe craniofacial features, and an increase in diameter >0.5 cm/year.

VSAR was initially introduced for patients with aortic root aneurysm having normal or minimal pathologic change of the aortic valve. Indications for VSAR in patients with significant AR remain controversial, since preoperative AR has been reported as a risk factor for recurrent AR. Previous literatures have described several aortic valve repair techniques with or without VSAR. Tanaka *et al.* [5] reported that free-margin reinforcement and central plication combined with VSAR enhanced the durability of the aortic valve, and use of patch was a risk factor for recurrent AR.

Most reports of aortic valve repair with VSAR were adult cases, though successful experiences with small children were extremely rare even in previous larger studies [1, 4]. VSAR and aortic valve repair are beneficial for small children, but long-term results regarding the durability of the aortic valve in children with LDS remains unknown. Indications for VSAR and aortic valve repair thus need to be considered with caution. We carefully inspected the size of fenestration, balance and strength of the aortic cusps intraoperatively and decided to perform aortic valve repair.

## CONCLUSION

We performed VSAR combined with aortic valve repair successfully, with no recurrent AR as of 5 years postoperatively. Frequent cardiovascular imaging of the whole arterial tree is necessary throughout life.

**Conflict of interest:** none declared. 

**Reviewer information:** Interactive CardioVascular and Thoracic Surgery thanks André Rüffer and the other, anonymous reviewer(s) for their contribution to the peer review process of this article.
